# Genetic Evolution of the Hemagglutinin Genes of Seasonal Influenza A Viruses in Türkiye Between 2017 and 2023

**DOI:** 10.1111/irv.70134

**Published:** 2025-06-27

**Authors:** M. Ekin Azbazdar, Mert Dikmenogullari, Zeynep Kavalci, Zeynep A. Koçer

**Affiliations:** ^1^ Department of Biomedicine and Health Technologies, Izmir International Biomedicine and Genome Institute Dokuz Eylul University Izmir Türkiye; ^2^ Emerging Viral Diseases Laboratory Izmir Biomedicine and Genome Center Izmir Türkiye

**Keywords:** antigenic variation, influenza A virus, natural selection, phylogenetic analysis, protein stability

## Abstract

**Background:**

Seasonal influenza A viruses (IAVs) remain a major global health concern, causing up to 650,000 deaths annually. Over the past century, four influenza pandemics have occurred, with H3N2 and H1N1 subtypes becoming endemic in humans. The hemagglutinin (HA) glycoprotein, essential for viral entry and a key vaccine target, contains critical antigenic sites. While antigenic drift enables immune evasion, certain substitutions can affect protein stability and intraprotein interactions, influencing viral fitness.

**Methods:**

This study employed a Bayesian approach to investigate the phylogenetic origins of full‐length HA genes from seasonal IAVs circulating in Izmir, Türkiye (2017–2023). Publicly available HA sequences from Türkiye were incorporated to assess selection pressures using four models available on Datamonkey and to examine antigenic mismatches between circulating viruses and vaccine strains. The structural impact of positively selected substitution was analyzed via molecular dynamics simulations.

**Results:**

Phylogenetic analysis identified four and six subclades for H1N1 and H3N2, respectively, revealing cocirculation of genetically distinct strains within the same season. Both subtypes were under negative selection, but the N260D substitution in H1N1 was consistently detected under positive selection across all models. Molecular dynamics simulations suggested that this substitution may influence intraprotein dynamics with the vestigial esterase domain, introducing a transient electrostatic bond. Furthermore, H3N2 exhibited more antigenic mismatches than H1N1, including a novel mismatch in 2022–2023.

**Conclusions:**

This is the first comprehensive study documenting the genetic evolution of IAVs in Türkiye over 6 years. Regional surveillance of antigenic changes can improve vaccine strain selection and vaccination strategies.

## 1 Introduction

Seasonal influenza A viruses (IAVs) pose a significant global health threat, causing an estimated 290,000–650,000 respiratory deaths annually [1]. Over the past century, IAVs have triggered four pandemics (1918, 1957, 1968, and 2009), resulting in an estimated death toll of over 50 million worldwide [2, 3, 4, 5]. Following the emergence of H3N2 in 1968 and H1N1 in 2009, viruses of these subtypes were established in humans and continue to evolve seasonally, presenting ongoing risk to public health.

The negative‐sense RNA genome of IAVs consists of eight gene segments that encode at least 10 proteins: polymerase basic 1 (PB1), PB2, polymerase acidic (PA), hemagglutinin (HA), nucleoprotein (NP), neuraminidase (NA), matrix 1 (M1), M2, nonstructural 1 (NS1), and NS2/NEP. Among these, the HA surface glycoprotein is critical for viral entry, mediating recognition and binding to sialic acid receptors on the host cell membrane [6]. Based on its well‐established structure, HA is divided into HA1 and HA2 subunits, which are further subdivided into receptor‐binding, vestigial esterase (VE), transmembrane, and fusion (F) subdomains. Upon entry into the host cell, precursor hemagglutinin (HA0) is cleaved by host cell proteases into HA1 and HA2, a process triggered by low pH in the endosome. This conformational change is essential for membrane fusion and is influenced by the acidic pH required for HA cleavage. Notably, the 110‐helix in the VE subdomain (HA1) interacts with the B‐loop of the F subdomain (HA2), which transitions into a helix during the conformational change, thus playing a critical role in protein stability [7]. Additionally, the VE subdomain serves as a target for several neutralizing antibodies, highlighting its dual role in immune evasion and protein stability [8].

HA glycoprotein, a primary influenza vaccine target, contains major antigenic sites on the HA1 subunit. H1 proteins include the Sa, Sb, Ca1, Ca2, and Cb epitopes, whereas H3 proteins harbor A, B, C, D, and E epitopes [9, 10, 11]. Due to the error‐prone nature of RNA‐dependent RNA polymerase and the selective pressures driven by the host immune system, the HA gene undergoes rapid evolution through antigenic drift. This process may allow certain variations to become dominant via natural selection, with viral evolution shaped by positive, neutral, or negative selection. Positive selection within antigenic sites may facilitate immune escape mutations and may lead to antigenic mismatches between circulating strains and vaccine strains, potentially reducing vaccine efficacy [12, 13, 14]. These substitutions can be associated with the antigenic properties of IAVs and can result in the emergence of genetically distinct strains between the seasons. For instance, while the 6B.1 subclade of H1N1 and the 3C.2a subclade of H3N2 were predominant during the 2017–2018 influenza season in the World Health Organization (WHO) European Region, the gradual accumulation of amino acid substitutions in the HA protein over five consecutive seasons facilitated the divergence of these strains into distinct subclades [15]. Consequently, the majority of H1N1 and H3N2 viruses circulating during the 2023–2024 influenza season are characterized by amino acid substitutions defining the 6B.1A.5a.2a and 3C.2a1b.2a.3a.1 subclades, respectively [16]. Therefore, WHO routinely monitors the emergence of genetically distinct strains and provides vaccine composition recommendations based on surveillance data from the Global Influenza Surveillance and Response System (GISRS).

Although positive selection may enhance viral fitness at specific antigenic sites, studies have indicated that neutral or negative selection can dominate when considering the entire HA protein, potentially preserving essential HA functions. For instance, from 1995 to 2005, H3N2 HA genes were under neutral selection, potentially leading to the extinction of coexisting lineages and the emergence of low‐frequency variants in subsequent seasons [17]. Similarly, studies from China (2009–2019) and India (2011–2021) revealed that the negative selection pressure in the HA gene of H1N1 viruses is potentially linked to incomplete and ongoing adaptation of IAVs in humans [18, 19].

Altogether, understanding the evolution of seasonal IAVs is essential for selecting vaccine strains that best represent current antigenic properties, thereby maximizing vaccine efficacy. In Türkiye, regular surveillance efforts are conducted, with viral gene sequences continuously uploaded to the EpiFlu database of the Global Initiative on Sharing All Influenza Data (GISAID) by the General Directorate of Public Health at the Ministry of Health. Building on these efforts, we analyzed the phylogenetic origins of full‐length HA genes of seasonal IAVs circulating in Izmir, the third most populous city in Türkiye, with a population exceeding four million and significant domestic and international tourist activity. With the inclusion of publicly available HA gene sequences from Türkiye on GISAID, we also conducted a comprehensive investigation into selection pressures and antigenic mismatches with vaccine strains for H1 and H3 viruses circulating between 2017 and 2023. Additionally, the N260D substitution (H1 numbering) in the HAs of H1 viruses was consistently identified as under positive selection across all models. While this substitution is characteristic of contemporary H1N1 viruses in the 6B.1A.5 subclade, the structural role has remained unclear in the literature [16]. Therefore, the potential role of this position was investigated through molecular dynamics simulations. To the best of our knowledge, this is the first study that comprehensively investigates the genetic and antigenic dynamics in the HA genes of seasonal IAVs in Türkiye.

## 2 Materials and Methods

### 2.1 Viruses

Retrospective IAV‐positive nasopharyngeal/oropharyngeal swab samples collected at Ege University Hospital between 2017 and 2023 were included in this study without any selection criteria or seasonal restrictions. IAVs were isolated in AX4 cells, a modified version of Madin–Darby canine kidney (MDCK) cells that overexpress human 2,6‐sialyltransferase (ST6Gal1), facilitating better viral replication for clinical isolates compared with standard MDCK cells [20]. AX4 cells were cultured in minimum essential medium (MEM; Gibco) supplemented with 7.5% sodium bicarbonate (2.25 mg/mL) (Gibco), 1× MEM amino acid solution (Gibco), 1× MEM vitamin solution (Sigma‐Aldrich), 2‐mM L‐glutamine (Gibco), 1× penicillin/streptomycin (Gibco), and 5% newborn calf serum (NCS; Gibco). Additionally, 2 μg/mL puromycin (Merck) was added to maintain the selection of the plasmid containing the ST6Gal1 gene. For viral infections, a medium was prepared with 4% bovine serum albumin instead of NCS. Cells were grown as monolayers in 75‐cm^2^ cell culture flasks or 35‐mm Petri dishes and maintained at 37°C in incubators with 5% CO_2_. Upon infection, cells were monitored for cytopathic effects for 24–72 h under a light microscope to confirm viral presence. All experiments were conducted in Biosafety Level‐2 laboratories.

### 2.2 HA Subtyping and Sequencing of Full‐Length HA

Viral RNAs were extracted from virus isolates using QIAamp Viral RNA Mini Kit (Qiagen), according to the manufacturers' instructions. Partial (634 bp) or full‐length HA genes were amplified using One‐Step RT‐PCR Kit (Qiagen). For partial amplification, HA‐1144 (5′‐GGAATGATAGATGGNTGGTAYGG‐3′) and Bm‐NS‐890R (5′‐ATATCGTCTCGTATTAGTAGAAACAAGGGTGTTTT‐3′) primers were used [21], allowing the amplification of different HA subtypes with one primer pair [21]. For full‐length H3 gene amplification, a widely used universal HA primer pair was employed: Bm‐HA‐1 (5′‐TATTCGTCTCAGGGAGCAAAAGCAGGGG‐3′) and Bm‐NS‐890R (5′‐ATATCGTCTCGTATTAGTAGAAACAAGGGTGTTTT‐3′) [22]. For full‐length H1 gene amplification, two overlapping primer sets recommended in the WHO guideline [23] were used: H1F1 (5′‐AGCAAAAGCAGGGGAAAATAAAAGC‐3′) and H1R1264 (5′‐CCTACTGCTGTGAACTGTGTATTC‐3′) for a 1264‐bp fragment, and HAFA (5′‐GGGAGAATGAACTATTACTGG‐3′) and HARend (5′‐AGTAGAAACAAGGGTGTTTTT‐3′) for a 979‐bp fragment. Each 50‐μL reaction mixture contained 4 μL of extracted RNA, 28 μL of nuclease‐free water, 1× RT‐PCR buffer, 400 μM of each dNTP, 2 μL of enzyme mix, and 0.6 μM of each primer. Thermal cycler conditions included reverse transcription at 50°C for 60 min, followed by denaturation at 95°C for 15 min, 40 cycles of 95°C for 1 min, 56°C for 1 min, and 72°C for 2 min, with a final extension at 72°C for 10 min. Amplicons were purified using either QIAquick Gel Extraction Kit (Qiagen) or QIAquick PCR Purification Kit (Qiagen), according to the manufacturers' instructions. Purified products were sent to a commercial facility (Macrogen) for Sanger sequencing using subtype‐specific primers (details are available upon request) for the H1 and H3 HA genes. Sequence reads were assembled using the reference‐based Sanger assembly module in Unipro UGENE (v48.0) [24], with A/California/07/2009 (H1N1) and A/Hong Kong/4801/2014 (H3N2) HA gene sequences as references. Low‐quality reads were trimmed by inspecting chromatograms, and each nucleotide position in the consensus sequence was ensured to be covered by at least two sequencing primers.

### 2.3 Selection of Representative HA Sequences

The genetic characteristics of the HA gene and the time to the most recent common ancestors (tMRCAs) of IAVs circulating in Izmir, Türkiye, between 2017 and 2023 were investigated using molecular clock phylogenetic analyses. Prior to analyses, global representative strains for both H1 and H3 subtypes were selected using a two‐step filtering process. Full‐length HA genes of the H1N1 and H3N2 viruses, along with metadata from clinical samples or cell culture isolates collected globally between September 2017 and March 2023, were retrieved from the GISAID database. Filtering was performed separately for each HA subtype. Duplicate sequences were removed as an initial step, with priority given to sequences from the clinical specimens.

In the first step, sequences with incomplete dates, nonnucleotide characters, and less than 1701 bp were eliminated and grouped by country and year, selecting one sequence randomly from each group using Augur (v25.1.1) implemented in Nextstrain [25]. Additionally, swine‐origin variant viruses were excluded from the H1N1 dataset to improve the resolution of the phylogenetic tree. The filtered sequences were aligned via MAFFT [26], implemented in Augur [25], based on the protein‐coding nucleotide sequences of reference strains (A/California/07/2009 for H1N1 and A/Hong Kong/4801/2014 for H3N2).

In the second step, the sequences were translated into amino acid sequences, and 100% identical sequences were clustered using the MMseqs2 program (v15‐6f452) [27], randomly selecting one sequence from each cluster. HA gene sequences of WHO‐recommended vaccine strains for the northern hemisphere between 2017 and 2023 were also retrieved from GISAID and included in the final representative virus datasets.

### 2.4 Molecular Clock Phylogeny

The HA clades and time to tMRCAs with 95% highest probability density (HPD) intervals were estimated using molecular clock phylogenies. These analyses employed the Bayesian Markov chain Monte Carlo (MCMC) approach implemented in BEAST (v10.5.0) [28], utilizing the BEAGLE Library (v4.0.0) [29], and incorporating sampling dates.

HA genes of IAVs from Izmir, Türkiye, were included in the global representative HA gene datasets, and the sequences were aligned using MAFFT [26], implemented in Augur [25]. Consequently, phylogenetic analyses were performed with 268 and 386 HA gene sequences for H1N1 and H3N2 viruses, respectively. To select the best‐fit nucleotide substitution model for each dataset, PartitionFinder 2 software (v2.1.1) [30] was used, choosing the best model according to the highest Bayesian information criterion (BIC). Accordingly, phylogenetic trees were constructed under the General Time‐Reversible nucleotide substitution model with invariant sites and a gamma distribution of rate variation across sites (GTR + G + I) [31, 32].

To identify the best‐fit clock and demographic models for each analysis, log marginal likelihoods for combinations of clock models (strict clock, uncorrelated lognormal relaxed clock, uncorrelated exponential relaxed clock, and random local clock) and demographic models (constant size, exponential growth, logistic growth, and expansion growth) were estimated using the generalized stepping‐stone (GSS) sampling method [33]. The model with the highest log marginal likelihood was selected (Table S2). For both H1 and H3 HA gene datasets, the strict clock with a constant‐size population was identified as the best‐fit model. Three independent MCMC runs were conducted for 100,000,000 generations, with sampling every 10,000 generations. Tree and log files were combined with a 10% burn‐in per run using LogCombiner (v10.5.0; BEAST package). Convergence and effective sample sizes (ESS) exceeding 200 for all parameters were assessed using Tracer (v1.7.2) [34]. Maximum clade credibility trees (MCCTs) were generated using TreeAnnotator (v10.5.0; BEAST package), and phylogenetic trees were visualized using FigTree (v1.4.4) (http://tree.bio.ed.ac.uk/software/figtree/). Phylogenetic trees were annotated with the continents where globally representative strains were selected in order to assess their geographic relatedness with the study population.

### 2.5 Genetic and Antigenic Evolution of HA

To investigate genetic and antigenic evolution in the H1 and H3 HA genes, sequences obtained in this study were aligned with publicly available full‐length HA gene sequences from Türkiye collected between September 2017 and March 2023, retrieved from GISAID. Alignments were performed using ClustalW in Unipro UGENE (v48.0) [24]. Before alignment, sequences with nonnucleotide characters and less than 1701 bp were eliminated using Augur (v25.1.1) implemented in Nextstrain [25]. Amino acid positions under positive or negative selection, along with the ratio of nonsynonymous to synonymous substitutions (dN/dS), in the HAs of H1 and H3 subtypes were assessed using following tools available in Datamonkey [35]: Mixed Effects Model for Episodic Diversifying Selection (MEME) [36], Single Likelihood Ancestor Counting (SLAC) [37], A Fast, Unconstrained Bayesian Approximation for Inferring Selection (FUBAR) [38], and Fixed Effects Likelihood (FEL) [37]. Whereas all four models identify sites under positive selection, only SLAC, FUBAR, and FEL detect positions under negative selection. The ratios of dN/dS were only calculated using MEME and SLAC, and the mean dN/dS values from the two models were calculated separately for each HA subtype. For MEME, SLAC, and FEL, a *p* value threshold of ≤ 0.05 was applied, whereas for FUBAR, a posterior probability threshold of > 0.95 was used.

To identify antigenic mismatches, all sequences, including those from Izmir and Türkiye, were aligned with the recommended vaccine strains for the corresponding influenza seasons. Antigenic differences were compared with both cell‐based and egg‐based vaccine strains, if recommended by the WHO for any season. Antigenic sites of H1N1 and H3N2 viruses were analyzed separately for each season based on the antigenic sites identified in the literature [9, 10, 11]. The frequency of antigenic mismatches at each position was calculated by dividing the number of sequences containing the substitution by the total number of sequences analyzed for that season.

### 2.6 Protein Modeling and Molecular Dynamics Simulations

To investigate the potential structural impact of the amino acid substitution identified in the selection analyses, whose frequency gradually increased from 2017 to 2023, consensus HA sequences from the 2017–2018 and 2022–2023 influenza seasons in Türkiye were extracted, modeled, and evaluated for amino acid interactions. HA sequences were first grouped by season and aligned using MAFFT in Unipro UGENE (v48.0) [24]. Consensus sequences for each season were exported, ensuring that all observed amino acid variations in the consensus sequences were present in at least one virus sequence. This step ensured the biological relevance of the constructed sequences. The HA proteins were modeled using a homology modeling approach, with the structure having the highest Global Model Quality Estimate (GMQE) score (PDB ID: 7KNA) selected via SWISS‐MODEL [39].

For molecular dynamics simulations, all ligands were removed from the protein structures prior to initiating the simulations, and the monomer structures were extracted from the modeled trimer structures to reduce the computational cost. TIP3P [40] water model was used for the solvation of the HA proteins, with the protein placed at the center of the system and at least 15 Å from the box edges. Both systems were ionized with 0.15‐M NaCl, and CHARMM36 force field parameters were implemented [41, 42, 43]. The systems were subjected to energy minimization for 40,000 steps, utilizing the conjugate gradient algorithm within the default minimizer of NAMD 2.14 [44]. Following the energy minimization, a 0.25‐ns equilibration was carried out under a canonical (NVT) ensemble, with the protein atoms positionally fixed. The same energy minimization and NVT equilibration procedure were repeated for the systems, without fixing the protein atoms. Subsequently, an isothermal–isobaric (NPT) equilibration was carried out for 10 ns at 310.15 K and 1 atm, applying Langevin piston pressure and Langevin dynamics temperature [45]. The production simulations were performed for 500 ns utilizing periodic boundary conditions and a timestep of 2 fs, with similar NPT ensembles.

To investigate the impact of the N260D substitution on the structure of H1 HA monomers in terms of protein flexibility, compactness, and stability, a comprehensive computational approach was employed. Root‐mean‐square deviation (RMSD) and root‐mean‐square fluctuation (RMSF) analyses, along with radius of gyration (Rg) and solvent‐accessible surface area (SASA) calculations, were performed using Visual Molecular Dynamics (VMD, v1.9.4) to assess overall structural changes and local fluctuations [46]. Intramolecular hydrogen bond and salt bridge formations were analyzed in VMD (v1.9.4) using the HBonds and Salt Bridges plugins, respectively, to evaluate the stability of internal interactions (hydrogen bond: cutoff distance = 3.5 Å, cutoff angle = 150°; salt bridge: cutoff distance = 3.2 Å). Dynamic cross‐correlation matrices (DCCMs) were generated using the Bio3D package in R to examine changes in correlated or anticorrelated motions upon substitution [47].

## 3 Results

### 3.1 Isolation Rate and Subtype Distribution

Among 192 retrospective nasopharyngeal/oropharyngeal swab samples processed in this study, a total of 103 virus isolates were obtained (H1 = 25, H3 = 78), corresponding to an overall isolation rate of 53.65% (103/192). The virus isolation rates were 27.27% (*n* = 6/22), 52.54% (*n* = 62/118), 78.26% (*n* = 18/23), and 58.62% (*n* = 17/29) during the 2017–2018, 2018–2019, 2021–2022, and 2022–2023 seasons, respectively (Figure S1). The full‐length HA genes were successfully amplified and sequenced for 70 virus isolates (H1 = 17 and H3 = 53), covering all seasons (Figure S1), while the yield of other amplicons (*n* = 33) was insufficient for sequencing. Due to the COVID‐19 pandemic, we were unable to obtain IAV‐positive samples between 2019 and 2021.

### 3.2 Genetically Diverse Influenza A Viruses Cocirculated in Izmir, Türkiye Within the Same Season

To investigate the phylogenetic relationships and tMRCAs of H1 (*n* = 17) and H3 (*n* = 53) viruses that circulated between 2017 and 2023 in Izmir, MCCT analyses were performed under an MCMC strict clock model, including vaccine strains and representative viruses with diverse genetic backgrounds and geographic origins across the world.

**FIGURE 1:**
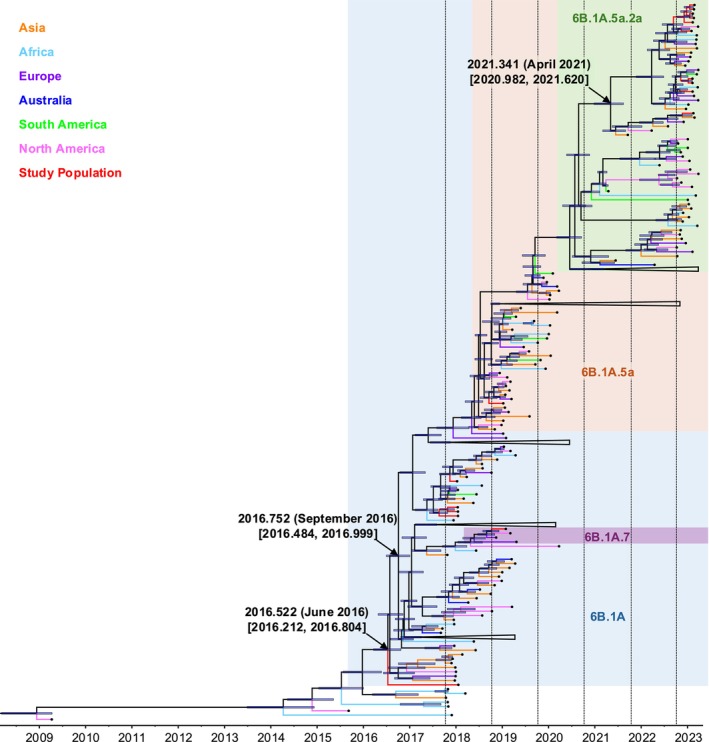
Maximum clade credibility tree for the H1 gene of IAVs in Izmir. Median times to tMRCAs with 95% HPD for selected nodes were marked with black arrows, and the 95% HPD of nodal divergence time was shown as blue bars. The scale bar represented years from 2009 to 2023, and dashed lines indicated the boundaries of influenza seasons. HA clades of the H1 viruses in the study population were depicted as different background colors: 6B.1A.5a.2a in green, 6B.1A.5a in salmon, 6B.1A.7 in purple, and 6B.1A in blue. Black dots marked the tip of each virus branch to improve branch visibility. Branches representing the study population were labeled in red, while branches representing viruses from other continents were color‐coded by region: Asia in orange, Africa in light blue, Europe in purple, Australia in dark blue, South America in green, and North America in pink. Viruses that did not exhibit a close phylogenetic relationship with those in the study population were collapsed to improve tree resolution.

The phylogenetic analysis showed that all H1 viruses from Izmir (*n* = 17) between 2017 and 2023 belonged to the clade 6B.1A, diverging into four subclades with tMRCA estimates around June 2016 (Figure 1). Viruses from the 2022–2023 season (*n* = 10) were found within the same subclade (6B.1A.5a.2a), with tMRCAs estimated to be around April 2021 (Figure 1). In contrast, viruses from the 2018–2019 season clustered into two different subclades (6B.1A.7: *n* = 1 and 6B.1A.5a: *n* = 1), with tMRCAs occurring around September 2016, suggesting concurrent circulation of genetically distinct variants in that season (Figure 1). Lastly, viruses from the 2017–2018 season (*n* = 5) were within the broader 6B.1A subgroup and showed genetic similarity to different representative strains compared with other seasons, with tMRCAs occurring around June 2016 (Figure 1).

Potential geographical relationships between the viruses in the study population and those in other continents were also evaluated. Among the 2022–2023 viruses in the subclade 6B.1A.5a.2a, six were genetically closer to the representative strains from North America and Africa, while three and one were genetically similar to the viruses from Africa, Europe, and South America and Asia, respectively (Figure 1). For the 2018–2019 season, one virus in the subclade 6B.1A.7 was genetically similar to a representative virus from North America, whereas another in the 6B.1A.5a subclade clustered with viruses from Europe, Asia, and North America (Figure 1). No specific clustering pattern was observed for the 2017–2018 viruses (Figure 1).

**FIGURE 2:**
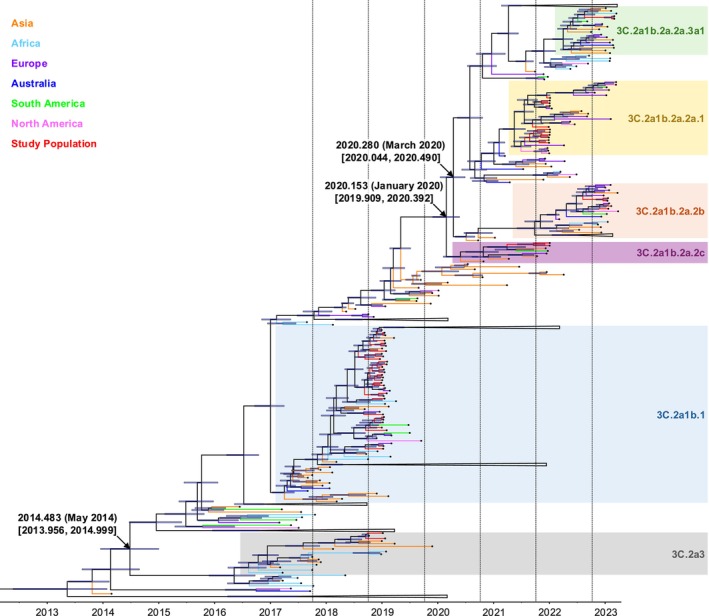
Maximum clade credibility tree for the H3 gene of IAVs in Izmir. Median times to tMRCAs with 95% HPD for selected nodes were marked with black arrows, and the 95% HPD of nodal divergence time was shown as blue bars. The scale bar represented years from 2013 to 2023, and dashed lines indicated the boundaries of influenza seasons. HA clades of the H3 viruses in the study population were depicted as different background colors: 3C.2a1b.2a.2a.3a1 in green, 3C.2a1b.2a.2a.1 in yellow, 3C.2a1b.2a.2b in salmon, 3C.2a1b.2a.2c in purple, 3C.2a1b.1 in blue, and 3C.2a3 in gray. Black dots marked the tip of each virus branch to improve branch visibility. Branches representing the study population were labeled in red, while branches representing viruses from other continents were color‐coded by region: Asia in orange, Africa in light blue, Europe in purple, Australia in dark blue, South America in green, and North America in pink. Viruses that did not exhibit a close phylogenetic relationship with those in the study population were collapsed to improve tree resolution.

All H3 viruses from Izmir between 2018 and 2023 were within the clade 3C.2a and diverged into six subclades, with tMRCA estimates around May 2014 (Figure 2). During the 2022–2023 season (*n* = 6), H3 viruses clustered into two subclades: 3C.2a1b.2a.2b (*n* = 4) and 3C.2a1b.2a.2a.3a1 (*n* = 2), with tMRCAs occurring around March 2020 (Figure 2). A similar pattern was observed during the 2021–2022 season. Some viruses (*n* = 11) were clustered within the same subclade (3C.2a1b.2a.2a.1), while others (*n* = 2) were within the 3C.2a1b.2a.2c subclade, with tMRCAs around January 2020 (Figure 2). In the 2018–2019 season, the majority of H3 viruses (*n* = 32) were part of the 3C.2a1b.1 subclade, whereas two strains clustered in the 3C.2a3 subclade. These results indicated that genetically distinct strains were cocirculated within the 2022–2023, 2021–2022, and 2018–2019 seasons (Figure 2).

The genetic relationships between H3 viruses in Izmir and representative strains were also investigated. In the 2022–2023 season, a virus from the 3C.2a1b.2a.2a.3a1 subclade was genetically similar to strains from Africa and Asia, whereas those in the 3C.2a1b.2a.2b subclade (*n* = 4) were genetically similar to viruses from Asia and Europe (Figure 2). During the 2021–2022 season, the viruses (*n* = 11) within the 3C.2a1b.2a.2a.1 subclade were genetically similar to strains from Europe, whereas the remaining two viruses from the 3C.2a1b.2a.2c subclade were clustered with the representative strains from Asia and South America (Figure 2). Lastly, the viruses in the 3C.2a1b.1 subclade (*n* = 32) clustered with strains from Asia, Europe, Africa, and South America, while two viruses in the 3C.2a3 subclade were genetically similar to strains from Asia and Africa (Figure 2).

### 3.3 Influenza A Viruses in Türkiye Were Under Negative Selection Pressure, but Position 260 in the H1 Protein Was Positively Selected

In addition to the clade identification, the antigenic evolution in the HAs of H1 and H3 viruses was assessed separately, using the sequences obtained in this study along with the publicly available ones for Türkiye on GISAID for the period of 2017–2023. Positive or negative selection was investigated to identify positions under selection in the HAs of H1 and H3 viruses. Four selection models (MEME, SLAC, FUBAR, and FEL) were used, focusing on positions with statistically significant *p* values (≤ 0.05) or posterior probabilities above 0.95. The dN/dS ratio was used to evaluate the type of selection that occurred during the evolution of HA genes. Values greater than one indicate positive selection, whereas values less than one suggest purifying (negative) selection.

The dN/dS ratios for HAs of viruses in the study population were calculated using two different models (MEME and SLAC). Because the FUBAR and FEL methods could not calculate these values, they were not included in the analyses. The average dN/dS values for H1 and H3 viruses were 0.2785 and 0.27, respectively, suggesting that the evolution of H1 and H3 subtypes in Türkiye between 2017 and 2023 was comparable, with both subtypes under purifying selection pressure (Table 1).

**TABLE 1:** Significantly important positions under positive (diversifying) or negative (purifying) selection in the HAs of H1 and H3 viruses in Türkiye between 2017 and 2023.

Subtype	Method	Position(s) under positive selection^a^, ^b^	Positions under negative selection^a^, ^b^	dN/dS
H1 (*n* = 173)	MEME^c^, ^e^	120, 260	N/A	0.268
	SLAC^c^, ^f^	260	30, **70**, 89, **124**, 147, 148, **168**, 290, 307, 334, 349, 385, 405, 431, 437, 472, 480, 510, 523	0.289
	FUBAR^d^, ^g^	120, 129, **189**, 256, 260	15, 30, 31, 33, **70**, 89, 100, 113, 115, **124**, 147, 148, 151, **168**, 179, 180, 211, **221**, **235**, 281, 290, 298, 299, 301, 307, 316, 323, 334, 343, 349, 358, 364, 373, 385, 400, 405, 417, 423, 431, 437, 462, 463, 472, 474, 480, 502, 510, 523, 525	N/A
	FEL^c^, ^h^	260	15, 30, 31, 61, **70**, 89, 100, 113, 115, **124**, 147, 148, 151, **168**, 179, 180, 211, **221**, 281, 290, 298, 299, 301, 307, 316, 323, 334, 343, 349, 358, 364, 373, 385, 405, 417, 423, 431, 437, 462, 463, 472, 474, 480, 502, 510, 523, 525	N/A
H3 (*n* = 186)	MEME^c^, ^e^	**156**	N/A	0.246
	SLAC^c^, ^f^	—	41, 148, 165, **196**, 325, 336, 359, 424, 433, 452, 463, 503	0.274
	FUBAR^d^, ^g^	**53**, **135**, **160**, **198**	24, 41, **44**, **45**, 75, **81**, **90**, 107, 109, 147, 148, 151, 161, 165, 181, **196**, **197**, **204**, **263**, **277**, 313, 325, 333, 336, 351, 352, 359, 367, 386, 407, 424, 425, 433, 436, 438, 452, 463, 488, 503	N/A
	FEL^c^, ^h^	**198**	41, **45**, **81**, **90**, 107, 109, 147, 148, 161, 165, 181, **196**, **197**, **204**, **263**, **277**, 313, 325, 333, 336, 351, 352, 357, 359, 367, 386, 407, 418, 424, 425, 433, 436, 438, 452, 463, 481, 488, 503	N/A

^a^ Antigenic sites were shown in bold.

^b^ Positions in the HAs of H1 and H3 viruses were indicated according to H1 and H3 numbering, respectively.

^c^ *p* values ≤ 0.05 were considered statistically significant.

^d^ Positions with a posterior probability of ≥ 0.95 were considered significant.

^e^ MEME: Mixed Effects Model for Episodic Diversifying Selection.

^f^ SLAC: Single Likelihood Ancestor Counting.

^g^ FUBAR: A Fast, Unconstrained Bayesian Approximation for Inferring Selection.

^h^ FEL: Fixed Effects Likelihood.

The positive selection pressure in the HA genes was further investigated using all four selection models (MEME, SLAC, FUBAR, and FEL). For both H1 (*n* = 173) and H3 (*n* = 186) viruses, positive selection in positions was limited, and no amino acid substitutions in the antigenic sites were found in any of the four models (Table 1). However, for H1 viruses, the N260D substitution, although not located in any antigenic site, was agreed by all four (MEME, SLAC, FUBAR, and FEL) selection models (Table 1).

Negative selection pressure in the HA genes of H1 and H3 viruses was more predominant compared with positions under positive selection, as analyzed using the SLAC, FUBAR, and FEL selection models. Because the MEME method could not calculate positions under negative selection, it was not included in the analyses. According to all three models, for H1 viruses, negative selection was identified at 19 positions, three of which (70, 124, and 168) were located within antigenic sites (Table 1). Similarly, for H3 viruses, 12 positions were determined to be under negative selection pressure based on all three models, with only one position (196) located in an antigenic site (Table 1).

### 3.4 H3N2 Viruses Exhibited Greater Antigenic Variations Than H1N1 Viruses Against Vaccine Strains

In addition to selection analyses, all HA sequences were compared with the vaccine strains of each corresponding season to identify antigenic mismatches. The HA sequences of the cell‐based vaccine strains were also included, if recommended by the WHO for any season. No sequence was available from Türkiye on GISAID for H1 HA in 2020–2021 and 2021–2022 and for H3 HA in 2019–2020 and 2020–2021. Therefore, 2022–2023 was considered the following season for both subtypes.

**FIGURE 3:**
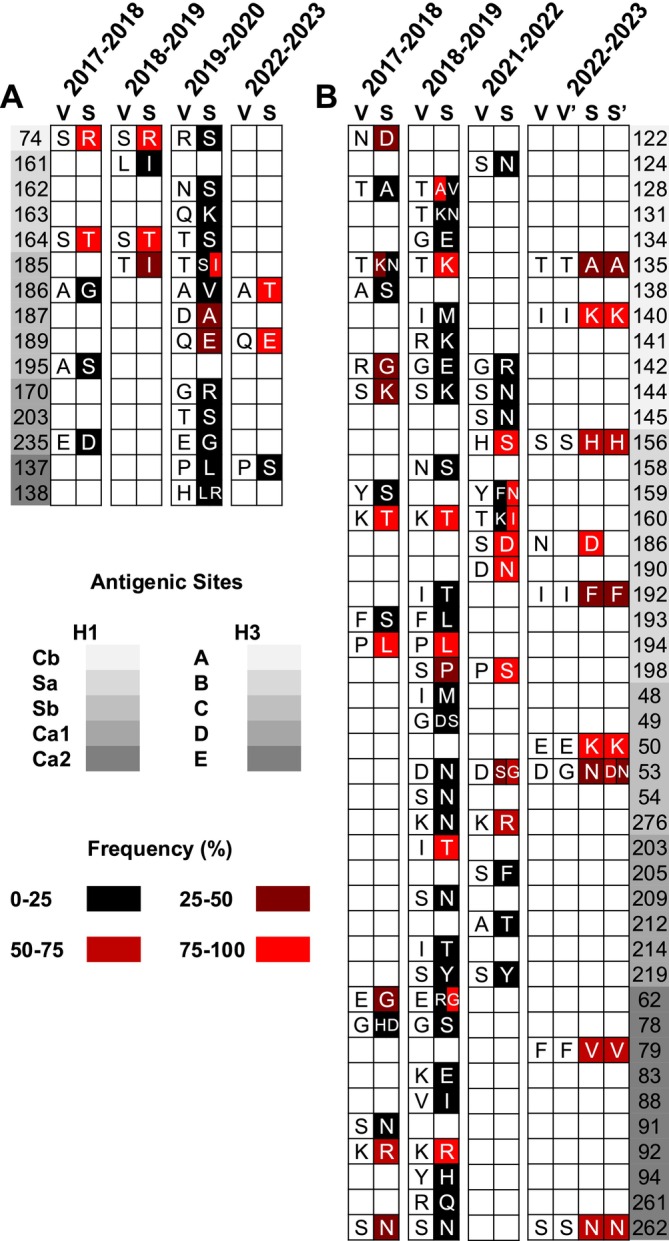
Antigenic mismatches of H1 and H3 viruses in Türkiye against vaccine strains. Antigenic mismatches in the HAs of H1 (A) and H3 (B) IAVs circulating in Türkiye between 2017 and 2023 were depicted in color scaling based on their frequencies (0 to 100%: from black to light red). The variations in the HA proteins of IAVs in the study population, where antigenic mismatch was observed, were compared with egg‐based (S for study population and V for vaccine strain) or cell‐based (S′ for study population and V′ for vaccine strain) vaccine strains. The amino acid positions and antigenic sites in the H1 (Cb, Sa, Sb, Ca1, and Ca2) and H3 (A, B, C, D, and E) proteins were listed on the left and right sides of the substitution panels, respectively, with the shades of gray. Positions in the HAs of H1 and H3 viruses were indicated according to H1 and H3 numbering, respectively.

In the 2017–2018 season, five antigenic mismatches were identified in the H1 HA protein compared with the vaccine strain, two of which (S74R and S164T) persisted in the 2018–2019 season (Figure 3A). Interestingly, during the 2019–2020 season, the S74R and S164T substitutions reverted to the amino acids of the vaccine virus recommended for the previous season. These reversions were observed at low frequencies and were no longer detected during the 2022–2023 season (Figure 3A and Table S3). Apart from positions 74 and 164, two additional antigenic mismatches were identified in the 2018–2019 season, with the mismatch at position 185 persisting into the following season (Figure 3A). In 2019–2020, 13 antigenic mismatches were observed, with the majority occurring at a low frequency (< 25%), eight of which were detected for the first time during the study period (Figure 3A and Table S3). Finally, in the 2022–2023 season, antigenic mismatches were identified in three positions, all of which were observed in previous seasons (Figure 3A).

Compared with H1 viruses, a higher number of antigenic mismatches was observed in the HAs of H3 viruses. In the 2017–2018 season, antigenic mismatches were identified at 15 positions with varying frequencies, 11 of which persisted into the 2018–2019 season (Figure 3B and Table S3). Notably, antigenic mismatches at three positions (142, 144, and 160) were observed across three consecutive seasons but were no longer detected in the 2022–2023 season (Figure 3B). In 2018–2019, 31 antigenic mismatches were observed, the majority (*n* = 23) of which were present at low frequencies (< 25%) in the study population (Figure 3B and Table S3). Position 53 was the only site that exhibited antigenic variation over the next three consecutive seasons, with the remaining stable or increasing frequency (Figure 3B and Table S3). Antigenic mismatches were identified at positions 15 and 9 during the 2021–2022 and 2022–2023 seasons, respectively. In 2022–2023, the mismatch at position 186 was identified only against the egg‐based vaccine strain, while two novel antigenic variations were observed at positions 50 and 79 (Figure 3B).

### 3.5 Asparagine‐to‐Aspartic Acid Substitution at Position 260 in the H1 Protein May Increase Protein Stability

Although the N260D substitution in H1 was not located within any known antigenic site, it was the only position identified as being under positive selection pressure by all four selection models (MEME, SLAC, FUBAR, and FEL) (Table 1). To assess its structural impact, consensus HA sequences of IAVs circulating during the 2017–2018 (HA‐N260) and 2022–2023 (HA‐D260) seasons in Türkiye were extracted, aligned, and modeled.

**FIGURE 4:**
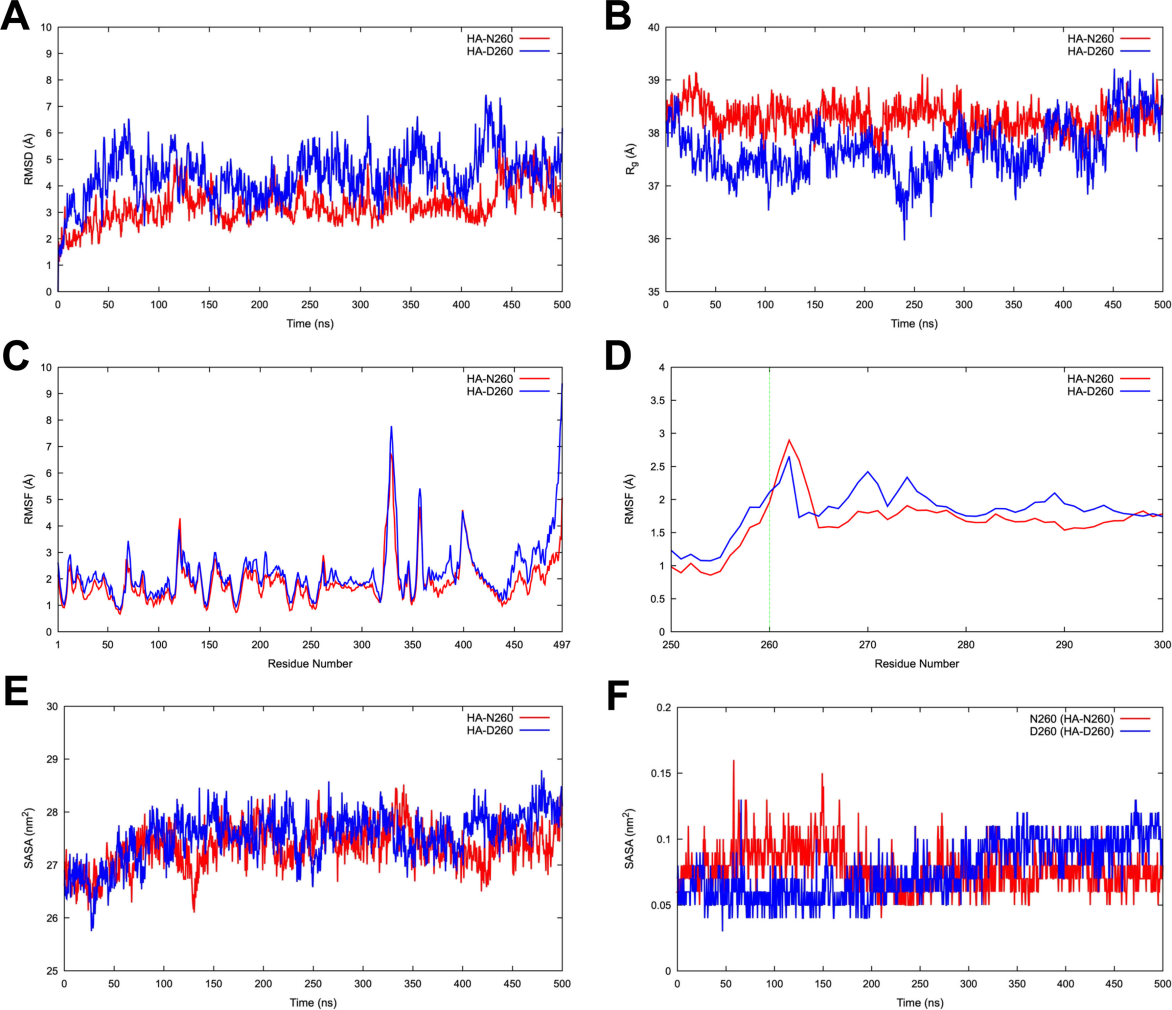
Structural and dynamic analyses of HAs of IAVs circulating during the 2017–2018 and 2022–2023 seasons. RMSD of backbone atoms over 500 ns (A) and Rg overtime (B) were calculated for both HA proteins. RMSF was assessed for the entire monomer structures (C) and specifically for residues 250–300 (D), with position 260 highlighted by the green vertical dashed line. Similarly, SASA was calculated for both overall structures (E) and specifically at position 260 (F).

First, the overall structural deviation between HA‐N260 and HA‐D260 was evaluated using RMSD calculations for the alpha carbon atoms. The results indicated that after an initial rise during the first 1–100 ns, RMSD values plateaued, suggesting that the system had reached equilibrium (Figure 4A). However, persistent fluctuations beyond this period indicated that both monomers continued to undergo minor conformational changes (Figure 4A). Notably, HA‐D260 exhibited noticeable fluctuations, suggesting an increased overall conformational flexibility (Figure 4A).

This trend was further supported by Rg calculations, which assessed structural stability and folding behavior. The results indicated that HA‐D260 exhibited slightly greater fluctuations than HA‐N260, implying that the N260D substitution may have contributed to increased global flexibility or conformational changes that influenced overall protein structure (Figure 4B). To investigate local structural flexibility, RMSF calculations were performed for both the entire monomer structure and specifically at position 260. In both monomers, the regions spanning residues 100–150 and 300–497 exhibited the highest flexibility; however, fluctuations were generally more pronounced in HA‐D260 (Figure 4C). Furthermore, the RMSF values at position 260 were comparable between HA‐N260 and HA‐D260, indicating that the N260D substitution did not induce global destabilization or significantly alter local residue flexibility (Figure 4D). Importantly, SASA analyses, which assessed structural stability, folding, and potential binding site exposure, for both the entire monomer structure (Figure 4E) and specifically at position 260 (Figure 4F), revealed lower fluctuations in HA‐D260 compared with HA‐N260. This suggested that while HA‐D260 exhibited increased global flexibility, its solvent‐exposed regions remained more stable, maintaining structural integrity. The consistency between Rg, RMSF, and SASA results suggested that the N260D substitution enhanced overall conformational adaptability without introducing major destabilizing effects.

**FIGURE 5:**
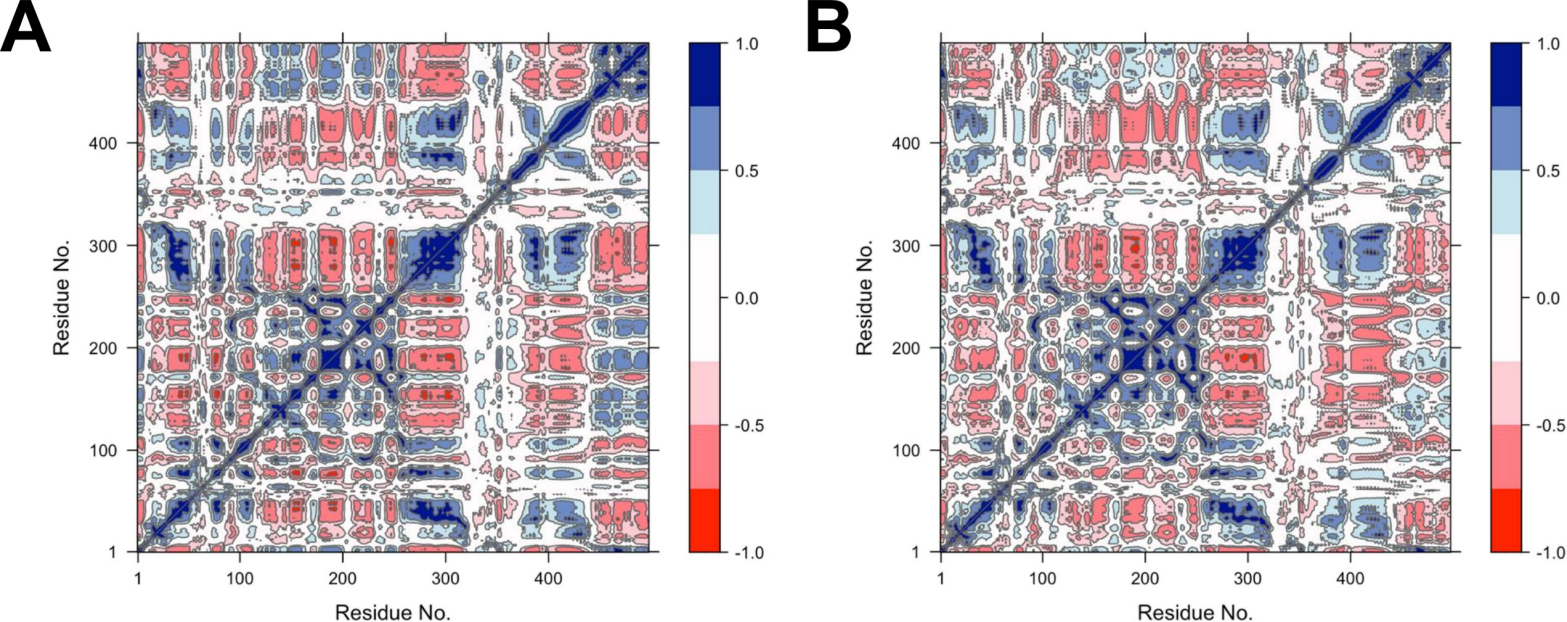
DCCM plots for HA monomers of IAVs circulating during the 2017–2018 and 2022–2023 seasons. Correlated residue–residue motions were analyzed for HA‐N260 (A) and HA‐D260 (B). The color scale represented the degree of correlation, with blue indicating strong positive correlation (coordinated motion in the same direction) and red representing strong negative correlation (opposing motions). The residue numbers were indicated on the *x*‐ and *y*‐axes of each plot.

The impact of the N260D substitution on residue–residue dynamics was further examined using DCCM analyses, which evaluated correlated and anticorrelated collective motions between residue 260 and other residues (Figure 5). The results showed that, with a correlation threshold of 0.7, indicating strong correlation, N260 exhibited correlated motions with 27 residues, whereas D260 showed correlated motions with 22 residues (Figure 5). The main differences for N260 were correlations with residues 49, 75, 79, 109, 171, 172, 257, 262, and 265 (Figure 5A), while D260 displayed distinct correlated motions with residues 44, 45, 46, and 276 (Figure 5B) (raw data are available upon request). These differences suggested that while the N260D substitution may have reduced correlated motions in the regions around residues 70, 100, and 170, it may have enhanced coordinated movements within the 44–46 region. Additionally, no anticorrelated residues with a correlation coefficient of −0.7 or lower were observed for either monomer.

**FIGURE 6:**
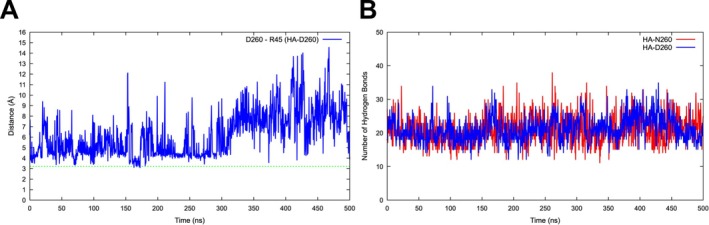
Salt bridge dynamics and hydrogen bond analysis at position 260 in HA monomers during molecular dynamics simulations. Salt bridge distance fluctuations (A) for the HA of IAVs circulating during the 2022–2023 season and hydrogen bond variations (B) for the HAs of IAVs circulating during the 2017–2018 and 2022–2023 seasons were analyzed over 500 ns of simulation. The green dashed line at 3.2 Å represented the threshold for salt bridge formation.

Because the introduction of a negatively charged aspartate at position 260 may alter electrostatic interactions, the potential structural impact of N260D was further investigated by analyzing salt bridges and hydrogen bonds, which contribute to protein stability and structural integrity. Our analyses indicated that a transient salt bridge was formed exclusively between D260 and R45, whereas no such interaction was observed for N260. In HA‐D260, aspartate substitution enabled the formation of a dynamic salt bridge with R45, fluctuating between 3 and 15 Å, and was observed only between 160 and 180 ns during the simulation (Figure 6A). Although not stable, its transient formation may have introduced localized electrostatic effects that influenced protein dynamics. Furthermore, these effects did not disrupt the overall hydrogen bonding network centered on residue 260 (Figure 6B). The number of hydrogen bonds between residue 260 and its interacting residues remained nearly identical after the substitution, with mean values of 20.88 ± 4.09 for N260 and 21.18 ± 3.74 for D260 (Figure 6B) (raw data are available upon request).

## 4 Discussion

Seasonal IAVs pose a significant global health threat, causing up to 650,000 respiratory deaths annually and presenting an ongoing public health risk [1]. Therefore, this study aimed to investigate the phylogenetic relationships between viruses that circulated in Izmir between 2017 and 2023 and representative strains from around the world, to explore the genetic evolution of full‐length HA genes of seasonal IAVs that were prevalent in Türkiye during this period, and to assess the potential role of the N260D substitution, which was consistently identified under positive selection across all models, in the HA protein of H1N1 viruses.

To achieve these aims, IAVs were first isolated from retrospective nasopharyngeal and oropharyngeal swab samples that tested positive for IAV. The HA subtype of each virus was determined, and full‐length HA gene sequences were subsequently obtained. The subtype distribution of IAVs isolated in Izmir was consistent with the data published by the General Directorate of Public Health between 2017 and 2023 (Figure S2). Because clinical specimens were obtained without selection criteria and all available samples were included, the number of samples varied by season. Although isolation rates varied by season, all except 2017–2018 (27.27%) exceeded 50%, indicating efficient virus isolation from retrospective clinical samples. Seasonal differences in isolation rates may be attributed to factors such as sample size and viral load. Viral load likely played a major role in isolation success, as higher titers may have contributed to better isolation efficiency [48], which could also explain the insufficient HA amplicons obtained from some cell culture isolates [49].

After obtaining full‐length HA genes, phylogenetic relationships between viruses circulating in Izmir from 2017 to 2023 and representative strains with diverse genetic backgrounds from across the world were investigated via Bayesian analyses. In line with the country‐wide data (Figure S3), all H1 viruses that circulated in Izmir during this period belonged to the clade 6B.1 and diverged from tMRCA in June 2016 into four subclades. While H1 viruses from the 2017–2018 and 2022–2023 seasons were placed into the same subclades within their respective seasons, those from the 2018–2019 season clustered into distinct subclades. This finding highlighted the simultaneous circulation of genetically distinct strains in Izmir during two different seasons, despite the limited number of viruses analyzed. Furthermore, phylogenetic analysis showed that while the viruses from the 2017–2018 and 2018–2019 seasons shared a common ancestor dating back to June and September 2016, respectively, those from the 2022–2023 season traced back to a more recent ancestor around April 2021. This shift in divergence time likely resulted from the impact of the COVID‐19 pandemic, which disrupted global influenza transmission [50] and may have led to the emergence or introduction of genetically distinct strains in Izmir. Although H1 viruses from the 2022–2023 season belonged to the same subclade, they may have originated from viruses circulating on different continents, even though representative strains were selected randomly. This hypothesis was supported by the observation that H1 viruses from Izmir clustered with strains from different continents during the 2018–2019 season, suggesting possible introductions from diverse geographic origins. Publicly available data also confirmed the genetic variability of H1 viruses during the 2018–2019 season, as evidenced by the concurrent circulation of viruses from four subclades in Türkiye (Figure S3).

Consistent with the country‐wide data (Figure S3), all H3 viruses that circulated in Izmir between 2018 and 2023 belonged to the clade 3C.2a and diverged from tMRCA in May 2014. Upon divergence, H3 viruses clustered into six subclades during this period. In the 2018–2019, 2021–2022, and 2022–2023 seasons, H3 viruses from two distinct subclades cocirculated in Izmir, again indicating the simultaneous circulation of genetically distinct strains in a single season. As observed for H1 viruses, tMRCA estimates for H3 viruses also revealed a temporal shift in divergence patterns. While viruses from the 2018–2019 season shared a common ancestor around May 2014, those from the 2021–2022 and 2022–2023 seasons traced back to more recent ancestors, dated to January and March 2020, respectively. These findings support the observations made for H1 viruses, suggesting that the COVID‐19 pandemic may have played a role in altering the evolutionary trajectory of both subtypes by limiting the viral diversity and facilitating the spread of more recently emerged lineages [51]. Despite this variation in divergence times, nearly all H3 viruses, except for 11 belonging to the 3C.2a1b.2a.2a.1 clade, clustered with strains circulating in Asia, alongside viruses from other regions. Genetic analysis of H3N2 viruses from 2002 to 2007 suggested a source‐sink model, in which novel strains emerge in East and Southeast Asia and spread globally, with other regions acting as sinks [52]. Combined with the fact that approximately 60% of the global population resides in Asia, these findings supported the notion that IAVs circulating in this region may play a critical role in shaping the diversity of seasonal IAVs detected in Izmir, particularly those of the H3 subtype. Additionally, the cocirculation of three distinct H3 subclades in Türkiye during the 2018–2019 season supported the genetic diversity observed in publicly available sequence data (Figure S3). Regardless of the limited number of sequences in the study population and the random selection of representative strains, these results suggested that genetically distinct H3 viruses cocirculated in Izmir and may have originated from diverse geographic sources.

Although phylogenetic analyses revealed genetic relatedness between viruses in our dataset and representative strains, the inferred geographic origins of these viruses remain speculative. This is due to the use of filtered datasets for both H1N1 and H3N2, in which genetically identical sequences were clustered using 100% identity thresholds. Such filtering may have excluded identical sequences from distinct geographic regions, thereby limiting the resolution of geographic inference. Further Bayesian phylogeographic analyses are therefore needed to evaluate the precise viral diffusion patterns of seasonal IAVs in Türkiye.

To investigate the sequence‐based antigenic evolution of IAVs across Türkiye, all publicly available full‐length HA gene sequences from 2017 to 2023 on GISAID were included alongside the sequences obtained in this study. Selection analyses based on dN/dS ratios revealed that the full‐length HA genes of both H1 and H3 viruses were under negative selection pressure. As negative selection pressure in the HA genes is generally associated with incomplete and ongoing adaptation of IAVs [18, 19], this may suggest that both H1 and H3 genes remained conserved over the six‐year period. Because antigenic selection driven by immune pressure depends on the presence of antibodies produced through natural infection or vaccination, both of which can force the selection of immune escape variants, the absence of positive selection may indicate insufficient population‐level immune responses in Türkiye. This could be due to low influenza vaccination coverage or the effects of original antigenic sin [53]. Supporting this, influenza vaccination rates in Türkiye were relatively low based on estimates from the Turkish Statistical Institute: 3.3% in 2014, 2.6% in 2016, 2.2% in 2019, and 2.7% in 2022 [54]. This can be further supported by the observation that while none of the antigenic sites were under positive selection, three antigenic positions for H1 viruses and one for H3 viruses were consistently identified under negative selection by all three models.

On the other hand, the N260D substitution in H1N1 viruses, although not located within a known antigenic site, was consistently identified as being under positive selection across all four models. This substitution is a defining genetic marker of contemporary H1N1 viruses in the 6B.1A.5 subclade and may represent an important evolutionary adaptation at the genetic level. However, its structural role remains unclear in the literature. In silico analyses indicated that HA‐D260 shows slightly greater flexibility than HA‐N260, and the N260D substitution did not introduce major destabilizing effects but may have enhanced the conformational adaptability. Notably, DCCM analyses revealed that N260D primarily influenced correlated motions with residues 44–46, a region where a transient salt bridge formed between D260 and R45. Although this salt bridge was not stably maintained, it appeared to modulate local structural stability with the VE subdomain. The N260D substitution did not induce global instability but altered local conformational dynamics. Given that the VE subdomain interacts with the B‐loop of the F subdomain [7], which transitions into a helix during the conformational change, such transient interactions may influence intraprotein dynamics, potentially affecting the pH‐dependent activation of HA and overall protein stability. Furthermore, some swine IAVs belonging to the 1A.3.3.2 clade that circulated between 2020 and 2022 harbored N at position 260 [55]. Considering that contemporary seasonal H1N1 viruses originated from the swine populations, the N260D substitution might represent a key molecular change facilitating the adaptation of IAVs from swine to humans. Because swine can be regarded as an intermediate host in the shift of HA activation pH between avian and human IAVs [56], this residue may play a critical role in host adaptation. However, further in vitro and in vivo studies are needed to better understand the specific role of the N260D substitution in this process.

In addition to selection analyses, antigenic mismatches against vaccine strains were examined for each antigenic site in H1 and H3 viruses across seasons. For H1 viruses, most mismatches occurred at low frequencies and eventually disappeared from the study population, with the exception of those at positions 186 and 189. Notably, during the 2019–2020 season, two amino acid substitutions (S74R and S164T) reverted to those found in the vaccine virus from the previous season. However, these substitutions were observed at low frequencies and subsequently disappeared during the 2022–2023 season.

Antigenic mismatches were more frequent in H3 viruses compared with H1 viruses, although most mismatches in the H3 HA proteins also occurred at low frequencies. Positions 142, 144, and 160 exhibited antigenic variations over three consecutive seasons (2017–2022) at varying frequencies; however, these mismatches eventually disappeared from the study population. In contrast, position 53 showed consistent variation across three consecutive seasons (2018–2023), with stable or increasing frequencies. Additionally, two novel mismatches at positions 50 and 79 were detected in the 2022–2023 season for both egg‐ and cell‐based vaccine strains. Further investigations into these positions, along with other antigenic sites, may improve our understanding of antigenic mismatches in H3 viruses and aid in selecting the most appropriate vaccine strains.

This study had some limitations. First, the limited number of isolates from Izmir restricted the scope of our findings. Despite the limited HA sequences, our results showed a high correlation with ESS values exceeding 200, which were sufficient for interpreting the outcomes of the Bayesian analyses. Second, the limited number of available HA sequences on GISAID may have hindered comprehensive geographic representation across Türkiye. The sequences analyzed were primarily derived from routine sentinel surveillance conducted by the Ministry of Health, representing a subset of cities with active sentinel centers. Additionally, global IAV circulation drastically declined during the COVID‐19 pandemic [50], particularly impacting the 2019–2020 and 2020–2021 seasons. This led to a substantial decrease in sample size during those years, limiting the ability to assess HA gene evolution during the pandemic period. Consequently, analyses of the antigenic evolution of IAVs were based solely on the HA sequences obtained in this study and the available sequences on GISAID. Lastly, due to the clustering of identical sequences during dataset filtering, the inferred geographic origins of the viruses remain speculative despite observed genetic relatedness in phylogenetic analyses.

## 5 Conclusions

In conclusion, this study provided insights into the phylogenetic origins of the full‐length HA genes of seasonal IAVs circulating in Izmir between 2017 and 2023, as well as revealing the antigenic evolution of the HA proteins of H1 and H3 strains that circulated in Türkiye between 2017 and 2023, based on publicly available data. Our analyses revealed that genetically distinct strains cocirculated in Izmir, and the predominant selection pressure in the HA genes of IAVs in Türkiye was negative, suggesting that the current HA structure tends to be preserved while shaping the viral evolution. Notably, the N260D substitution in the HAs of H1 viruses, a key substitution in contemporary 6B.1A.5 subclade viruses, was consistently identified under positive selection. Structural analysis suggested that this substitution may impact HA stability by altering interactions with the VE subdomain.

To the best of our knowledge, this is the first study reporting the genetic evolution of seasonal IAVs in Türkiye for 6 years, including the appearance and disappearance of IAVs from the population due to COVID‐19 pandemic. Further such studies would be needed at global level to comprehend the changes in IAVs pre‐ and post‐COVID‐19 pandemic era. Monitoring genetic and antigenic changes of IAVs at regional level could also reveal regional differences in viral populations, as well as cocirculation of viruses from different origins, that would eventually help with the vaccination efforts in countries.

## Author Contributions

**M. Ekin Azbazdar:** conceptualization, investigation, writing – original draft, writing – review and editing, data curation, formal analysis, visualization. **Mert Dikmenogullari:** data curation, formal analysis, investigation, writing – review and editing, writing – original draft. **Zeynep Kavalci:** formal analysis, investigation, visualization, writing – original draft, writing – review and editing. **Zeynep A. Koçer:** conceptualization, funding acquisition, writing – original draft, writing – review and editing, project administration, supervision, resources, formal analysis, investigation, data curation.

## Ethics Statement

The virus isolation was performed from the retrospective nasopharyngeal/oropharyngeal swab samples obtained from the repository of Ege University Hospital between 2017 and 2023 with the permission of ethical committee approval obtained from the Medical Research Ethics Committee at Ege University (Approval Number: 20‐5.IT/49).

## Conflicts of Interest

The authors declare no conflicts of interest.

## Peer Review

The peer review history for this article is available at https://www.webofscience.com/api/gateway/wos/peer‐review/10.1111/irv.70134.

## Supporting information


**Table S1.** The list of H1 and H3 viruses in Izmir, Türkiye between 2017 and 2023 with collection dates and NCBI accession codes.
**Table S2.** Log marginal likelihood results for selecting the appropriate clock and demographic models in Bayesian phylogenetic analyses of HA genes from H1 and H3 IAVs.
**Table S3.** Amino acid substitutions with frequencies in the antigenic sites of H1 and H3 viruses in Türkiye between 2017 and 2023.
**Figure S1.** Distributions of the total number of samples, isolates, and sequenced HAs for H1 and H3 strains in each season. Seasons were color‐coded: 2017–2018 in black, 2018–2019 in yellow, 2021–2022 in light blue, and 2022–2023 in salmon.
**Figure S2.** Subtype distribution of IAVs in Türkiye. The H1 and H3 subtype distribution of IAVs in Türkiye between 2017–2023 was obtained from the publicly available weekly influenza reports that were prepared by the General Directorate of Public Health, Ministry of Health in Turkish (https://grip.saglik.gov.tr/tr/haftalik‐influenza‐raporu). Since no IAV data were available for the 2019–2020 season, the corresponding column was left empty. Subtypes were color‐coded: H3N2 in orange and H1N1 in blue.
**Figure S3.** HA clade distribution of IAVs in Türkiye. The HA clades of H1 (A) and H3 (B) IAVs in Türkiye between 2017–2023 were extracted from publicly available metadata obtained from GISAID. Clades were color‐coded: unassigned in orange, 6B.1 in black, 6B.1A in dark blue, 6B.1A.2 in pink, 6B.1A.7 in light gray, 6B.1A.5b in salmon, 6B.1A.5a in light blue, 6B.1A.5a.1 in light brown, and 6B.1A.5a.2a in dark gray for H1N1 viruses, unassigned in orange, 3C.3a in dark blue, 3C.2a3 in pink, 3C.2a1 in light gray, 3C.2a1b.1 in salmon, 3C.2a1b.2 in light blue, 3C.2a1b.2a.2a.1 in light brown, and 3C.2a1b.2a.2a.3a.1 in dark gray for H3N2 viruses.

## Data Availability

The HA sequences were uploaded to NCBI GenBank with accession numbers between OM171280‐OM171340 and PP582341‐PP582379 (Table S1). The HA sequences of representative strains and viruses from Türkiye were retrieved from EpiFlu Database of GISAID (https://gisaid.org/).
