# Supplementary material for: Genetic Evolution of the Hemagglutinin Genes of Seasonal Influenza A Viruses in Türkiye Between 2017 and 2023
Source: Influenza Other Respir Viruses. 2025 Jun 27;19(7):e70134. doi: 10.1111/irv.70134 (PMC12203568; doi:10.1111/irv.70134)
Supplement: Supplementary file 1 — Table S1. The list of H1 and H3 viruses in Izmir, Türkiye between 2017 and 2023 with collection dates and NCBI accession codes. Table S2. Log marginal likelihood results for selecting the appropriate clock and demographic models in Bayesian phylogenetic analyses of HA genes from H1 and H3 IAVs. Table S3. Amino acid substitutions with frequencies in the antigenic sites of H1 and H3 viruses in Türkiye between 2017 and 2023. Figure S1. Distributions of the total number of samples, isolates, and sequenced HAs for H1 and H3 strains in each season. Seasons were color‐coded: 2017–2018 in black, 2018–2019 in yellow, 2021–2022 in light blue, and 2022–2023 in salmon. Figure S2. Subtype distribution of IAVs in Türkiye. The H1 and H3 subtype distribution of IAVs in Türkiye between 2017–2023 was obtained from the publicly available weekly influenza reports that were prepared by the General Directorate of Public Health, Ministry of Health in Turkish (https://grip.saglik.gov.tr/tr/haftalik‐influenza‐raporu). Since no IAV data were available for the 2019–2020 season, the corresponding column was left empty. Subtypes were color‐coded: H3N2 in orange and H1N1 in blue. Figure S3. HA clade distribution of IAVs in Türkiye. The HA clades of H1 (A) and H3 (B) IAVs in Türkiye between 2017–2023 were extracted from publicly available metadata obtained from GISAID. Clades were color‐coded: unassigned in orange, 6B.1 in black, 6B.1A in dark blue, 6B.1A.2 in pink, 6B.1A.7 in light gray, 6B.1A.5b in salmon, 6B.1A.5a in light blue, 6B.1A.5a.1 in light brown, and 6B.1A.5a.2a in dark gray for H1N1 viruses, unassigned in orange, 3C.3a in dark blue, 3C.2a3 in pink, 3C.2a1 in light gray, 3C.2a1b.1 in salmon, 3C.2a1b.2 in light blue, 3C.2a1b.2a.2a.1 in light brown, and 3C.2a1b.2a.2a.3a.1 in dark gray for H3N2 viruses. [file IRV-19-e70134-s001.docx]

**Table S1**. The list of H1 and H3 viruses in Izmir, Türkiye between 2017 and 2023 with collection dates and NCBI accession codes.

| Season | Subtype | Strain | Collection Date | NCBI Accession Codes |
| --- | --- | --- | --- | --- |
| 2017-2018  (n=5) | **H1**  **(n=5)** | A/Izmir/14/2018 | 2018-01-15 | PP582341 |
|  |  | A/Izmir/15/2018 | 2018-01-17 | PP582342 |
|  |  | A/Izmir/19/2018 | 2018-01-10 | PP582343 |
|  |  | A/Izmir/21/2018 | 2018-01-20 | PP582344 |
|  |  | A/Izmir/22/2018 | 2018-01-17 | PP582345 |
| 2018-2019  (n=36) | **H1**  **(n=2)** | A/Izmir/28/2019 | 2019-01-29 | PP582346 |
|  |  | A/Izmir/32/2019 | 2019-01-09 | PP582347 |
|  | **H3**  **(n=34)** | A/Izmir/01/2018 | 2018-12-19 | OM171280 |
|  |  | A/Izmir/02/2018 | 2018-12-28 | OM171282 |
|  |  | A/Izmir/03/2018 | 2018-12-31 | OM171284 |
|  |  | A/Izmir/04/2018 | 2018-12-30 | OM171286 |
|  |  | A/Izmir/06/2018 | 2018-12-31 | PP582358 |
|  |  | A/Izmir/01/2019 | 2019-01-02 | OM171288 |
|  |  | A/Izmir/02/2019 | 2019-01-03 | OM171290 |
|  |  | A/Izmir/03/2019 | 2019-01-03 | OM171292 |
|  |  | A/Izmir/04/2019 | 2019-01-07 | OM171294 |
|  |  | A/Izmir/05/2019 | 2019-01-07 | OM171296 |
|  |  | A/Izmir/06/2019 | 2019-01-08 | OM171298 |
|  |  | A/Izmir/07/2019 | 2019-01-09 | OM171300 |
|  |  | A/Izmir/08/2019 | 2019-01-09 | OM171302 |
|  |  | A/Izmir/09/2019 | 2019-01-10 | OM171304 |
|  |  | A/Izmir/10/2019 | 2019-01-11 | OM171306 |
|  |  | A/Izmir/11/2019 | 2019-01-12 | OM171308 |
|  |  | A/Izmir/12/2019 | 2019-01-14 | OM171310 |
|  |  | A/Izmir/13/2019 | 2019-01-14 | OM171312 |
|  |  | A/Izmir/14/2019 | 2019-01-18 | OM171314 |
|  |  | A/Izmir/15/2019 | 2019-01-18 | OM171316 |
|  |  | A/Izmir/16/2019 | 2019-01-18 | OM171318 |
|  |  | A/Izmir/17/2019 | 2019-01-18 | OM171320 |
|  |  | A/Izmir/18/2019 | 2019-01-21 | OM171322 |
|  |  | A/Izmir/19/2019 | 2019-01-22 | OM171323 |
|  |  | A/Izmir/20/2019 | 2019-01-22 | OM171325 |
|  |  | A/Izmir/21/2019 | 2019-01-23 | OM171327 |
|  |  | A/Izmir/22/2019 | 2019-01-27 | OM171329 |
|  |  | A/Izmir/23/2019 | 2019-01-28 | OM171331 |
|  |  | A/Izmir/24/2019 | 2019-02-01 | OM171333 |
|  |  | A/Izmir/25/2019 | 2019-02-01 | OM171335 |
|  |  | A/Izmir/26/2019 | 2019-02-01 | OM171337 |
|  |  | A/Izmir/27/2019 | 2019-02-04 | OM171339 |
|  |  | A/Izmir/30/2019 | 2019-01-14 | PP582359 |
|  |  | A/Izmir/34/2019 | 2019-01-08 | PP582360 |
| 2021-2022  (n=13) | **H3**  **(n=13)** | A/Izmir/02/2021 | 2021-12-28 | PP582361 |
|  |  | A/Izmir/03/2021 | 2021-12-30 | PP582362 |
|  |  | A/Izmir/04/2021 | 2021-12-28 | PP582363 |
|  |  | A/Izmir/15/2021 | 2021-12-29 | PP582364 |
|  |  | A/Izmir/03/2022 | 2022-01-03 | PP582365 |
|  |  | A/Izmir/04/2022 | 2022-01-04 | PP582366 |
|  |  | A/Izmir/05/2022 | 2022-01-04 | PP582367 |
|  |  | A/Izmir/07/2022 | 2022-01-04 | PP582368 |
|  |  | A/Izmir/10/2022 | 2022-01-03 | PP582369 |
|  |  | A/Izmir/11/2022 | 2022-01-03 | PP582370 |
|  |  | A/Izmir/12/2022 | 2022-01-04 | PP582371 |
|  |  | A/Izmir/14/2022 | 2022-01-05 | PP582372 |
|  |  | A/Izmir/16/2022 | 2022-01-06 | PP582373 |
| 2022-2023  (n=16) | **H1**  **(n=10)** | A/Izmir/01/2023 | 2023-02-12 | PP582348 |
|  |  | A/Izmir/02/2023 | 2023-02-17 | PP582349 |
|  |  | A/Izmir/03/2023 | 2023-02-21 | PP582350 |
|  |  | A/Izmir/04/2023 | 2023-02-22 | PP582351 |
|  |  | A/Izmir/05/2023 | 2023-02-26 | PP582352 |
|  |  | A/Izmir/09/2023 | 2023-02-11 | PP582353 |
|  |  | A/Izmir/10/2023 | 2023-02-13 | PP582354 |
|  |  | A/Izmir/11/2023 | 2023-02-10 | PP582355 |
|  |  | A/Izmir/12/2023 | 2023-02-10 | PP582356 |
|  |  | A/Izmir/13/2023 | 2023-02-02 | PP582357 |
|  | **H3**  **(n=6)** | A/Izmir/07/2023 | 2023-01-22 | PP582374 |
|  |  | A/Izmir/08/2023 | 2023-02-10 | PP582375 |
|  |  | A/Izmir/15/2023 | 2023-01-20 | PP582376 |
|  |  | A/Izmir/16/2023 | 2023-01-16 | PP582377 |
|  |  | A/Izmir/18/2023 | 2023-03-02 | PP582378 |
|  |  | A/Izmir/19/2023 | 2023-03-02 | PP582379 |

**Table S2.** Log marginal likelihood results for selecting the appropriate clock and demographic models in Bayesian phylogenetic analyses of HA genes from H1 and H3 IAVs.

| **Subtype** | **Clock Model** | **Demographic Model** | **Marginal Likelihood (log)** |
| --- | --- | --- | --- |
| **H1** | **Strict** | **Constant Size** | **-15425.024629060994** |
|  |  | Exponential Growth | -15432.170281471594 |
|  |  | Logistic Growth | -15491.945929043293 |
|  |  | Expansion Growth | -15427.801550293025 |
|  | Uncorrelated Relaxed (lognormal) | Constant Size | -15426.484559448016 |
|  |  | Exponential Growth | -15431.857412215904 |
|  |  | Logistic Growth | -15469.766546458332 |
|  |  | Expansion Growth | -15436.246330046188 |
|  | Uncorrelated Relaxed (exponential) | Constant Size | -15463.163541679396 |
|  |  | Exponential Growth | -15470.033429800034 |
|  |  | Logistic Growth | -15494.746837387253 |
|  |  | Expansion Growth | -15462.984078768268 |
|  | Random Local | Constant Size | -15426.931986942225 |
|  |  | Exponential Growth | -15430.141910220811 |
|  |  | Logistic Growth | -15458.538928685157 |
|  |  | Expansion Growth | -15428.456541494605 |
| **H3** | **Strict** | **Constant Size** | **-15854.581429630376** |
|  |  | Exponential Growth | -15863.004574991064 |
|  |  | Logistic Growth | -15869.043092336919 |
|  |  | Expansion Growth | -15869.354442090750 |
|  | Uncorrelated Relaxed (lognormal) | Constant Size | -15863.641131612083 |
|  |  | Exponential Growth | -15947.838169739833 |
|  |  | Logistic Growth | -15873.844466684957 |
|  |  | Expansion Growth | -15872.341641265650 |
|  | Uncorrelated Relaxed (exponential) | Constant Size | -15940.186086732348 |
|  |  | Exponential Growth | -15947.942939825280 |
|  |  | Logistic Growth | -15949.944461606221 |
|  |  | Expansion Growth | -15946.503587576708 |
|  | Random Local | Constant Size | -15857.296947969902 |
|  |  | Exponential Growth | -15861.333554357640 |
|  |  | Logistic Growth | -15866.921992822718 |
|  |  | Expansion Growth | -15867.821029372884 |

**Table S3**. Amino acid substitutions with frequencies in the antigenic sites of H1 and H3 viruses in Türkiye between 2017 and 2023.

| Subtype | Antigenic Site | Position | Substitutions by Season (% frequency) | | | | |
| --- | --- | --- | --- | --- | --- | --- | --- |
|  |  |  | **2017-2018**  **(n=94)** | **2018-2019**  **(n=161)** | **2019-2020**  **(n=68)** | **2021-2022**  **(n=17)** | **2022-2023^a^**  **(n=19)** |
| H1  (n=173) | **Cb** | 74 | S → R (97.40) | S → R (100) | R → S (2.94) |  |  |
|  | **Sa** | 161 |  | L → I (6.25) |  |  |  |
|  |  | 162 |  |  | N → S (5.88) |  |  |
|  |  | 163 |  |  | Q → K (5.88) |  |  |
|  |  | 164 | S → T (94.81) | S → T (100) | T → S (5.88) |  |  |
|  | **Sb** | 185 |  | T → I (37.50) | T → I/S (97.06/2.94) |  |  |
|  |  | 186 | A → G (1.30) |  | A → V (1.47) |  | A → T (100) |
|  |  | 187 |  |  | D → A (35.29) |  |  |
|  |  | 189 |  |  | Q → E (32.35) |  | Q → E (100) |
|  |  | 195 | A → S (1.30) |  |  |  |  |
|  | **Ca1** | 170 |  |  | G → R (1.47) |  |  |
|  |  | 203 |  |  | T → S (1.47) |  |  |
|  |  | 235 | E → D (1.30) |  | E → G (2.94) |  |  |
|  | **Ca2** | 137 |  |  | P → L (1.47) |  | P → S (8.33) |
|  |  | 138 |  |  | H → L/R (8.82/1.47) |  |  |
| H3  (n=186) | **A** | 122 | N → D (35.29) |  |  |  |  |
|  |  | 124 |  |  |  | S →N (5.88) |  |
|  |  | 128 | T → A (11.76) | T → A/V (97.24/0.69) |  |  |  |
|  |  | 131 |  | T → K/N (1.38/0.69) |  |  |  |
|  |  | 134 |  | G → E (0.69) |  |  |  |
|  |  | 135 | T → K/N (29.41/23.53) | T → K (97.24) |  |  | T → A (28.57)  Egg- and cell-based |
|  |  | 138 | A → S (11.76) |  |  |  |  |
|  |  | 140 |  | I → M (0.69) |  |  | I → K (100)  Egg- and cell-based |
|  |  | 141 |  | R → K (1.38) |  |  |  |
|  |  | 142 | R → G (41.18) | G → E (0.69) |  | G → R (11.76) |  |
|  |  | 144 | S → K (47.06) | S → K (2.76) |  | S → N (11.76) |  |
|  |  | 145 |  |  |  | S → N (11.76) |  |
|  | **B** | 156 |  |  |  | H → S (76.47) | S → H (57.14)  Egg- and cell-based |
|  |  | 158 |  | N → S (0.69) |  |  |  |
|  |  | 159 | Y → S (11.76) |  |  | Y → F/N (11.76/88.24) |  |
|  |  | 160 | K → T (88.24) | K → T (97.93) |  | T → I/K (88.24/11.76) |  |
|  |  | 186 |  |  |  | S → D (100) | N → D (100)  Egg-based |
|  |  | 190 |  |  |  | D → N (100) |  |
|  |  | 192 |  | I → T (0.69) |  |  | I → F (42.86)  Egg- and cell-based |
|  |  | 193 | F → S (11.76) | F → L (0.69) |  |  |  |
|  |  | 194 | P → L (100) | P → L (100) |  |  |  |
|  |  | 198 |  | S → P (63.45) |  | P → S (100) |  |
|  | **C** | 48 |  | I → M (2.07) |  |  |  |
|  |  | 49 |  | G → D/S (2.07/1.38) |  |  |  |
|  |  | 50 |  |  |  |  | E → K (100)  Egg- and cell-based |
|  |  | 53 |  | D → N (3.45) |  | D → G/S (58.82/29.41) | D → N (42.86)  Egg-based  G → D/N (57.14/42.86)  Cell-based |
|  |  | 54 |  | S → N (0.69) |  |  |  |
|  |  | 276 |  | K → N (1.38) |  | K → R (76.47) |  |
|  | **D** | 203 |  | I → T (100) |  |  |  |
|  |  | 205 |  |  |  | S → F (11.76) |  |
|  |  | 209 |  | S → N (0.69) |  |  |  |
|  |  | 212 |  |  |  | A → T (11.76) |  |
|  |  | 214 |  | I → T (1.38) |  |  |  |
|  |  | 219 |  | S → Y (0.69) |  | S → Y (5.88) |  |
|  | **E** | 62 | E → G (29.41) | E → G/R (96.55/0.69) |  |  |  |
|  |  | 78 | G → D/H (23.53/5.88) | G → S (0.69) |  |  |  |
|  |  | 79 |  |  |  |  | F → V (57.14)  Egg- and cell-based |
|  |  | 83 |  | K → E (1.38) |  |  |  |
|  |  | 88 |  | V → I (3.45) |  |  |  |
|  |  | 91 | S → N (11.76) |  |  |  |  |
|  |  | 92 | K → R (52.94) | K → R (87.59) |  |  |  |
|  |  | 94 |  | Y → H (1.38) |  |  |  |
|  |  | 261 |  | R → Q (4.83) |  |  |  |
|  |  | 262 | S → N (35.29) | S → N (2.07) |  |  | S → N (57.14)  Egg- and cell-based |

^a^The antigenic differences were compared against both cell- and egg-based vaccine strains, as two different strains were recommended by WHO for cell- or egg-based vaccine in the 2022-2023 season.

**
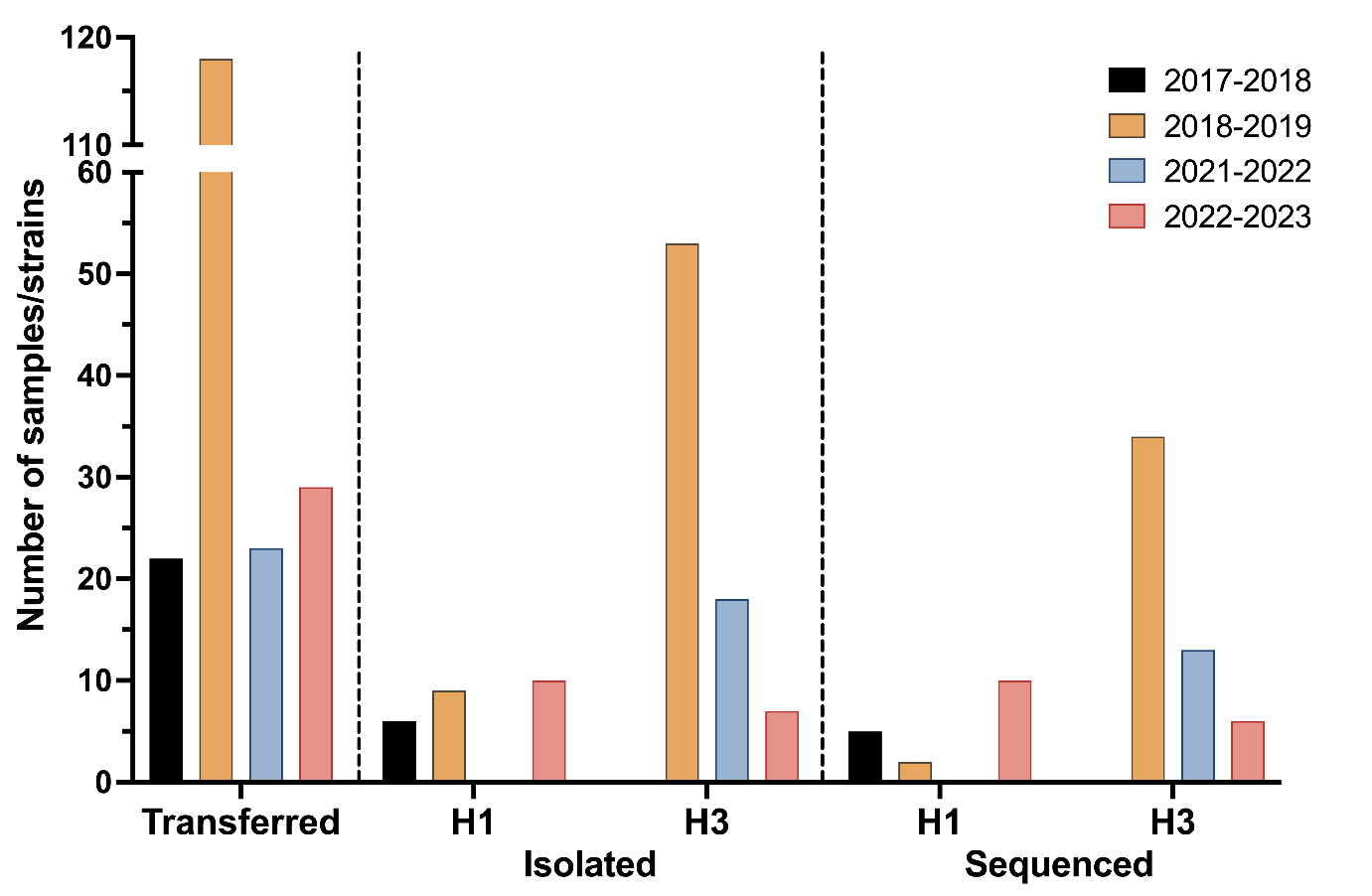
**

**Figure S1.** Distributions of the total number of samples, isolates, and sequenced HAs for H1 and H3 strains in each season. Seasons were color-coded: 2017-2018 in black, 2018-2019 in yellow, 2021-2022 in light blue, and 2022-2023 in salmon.

**
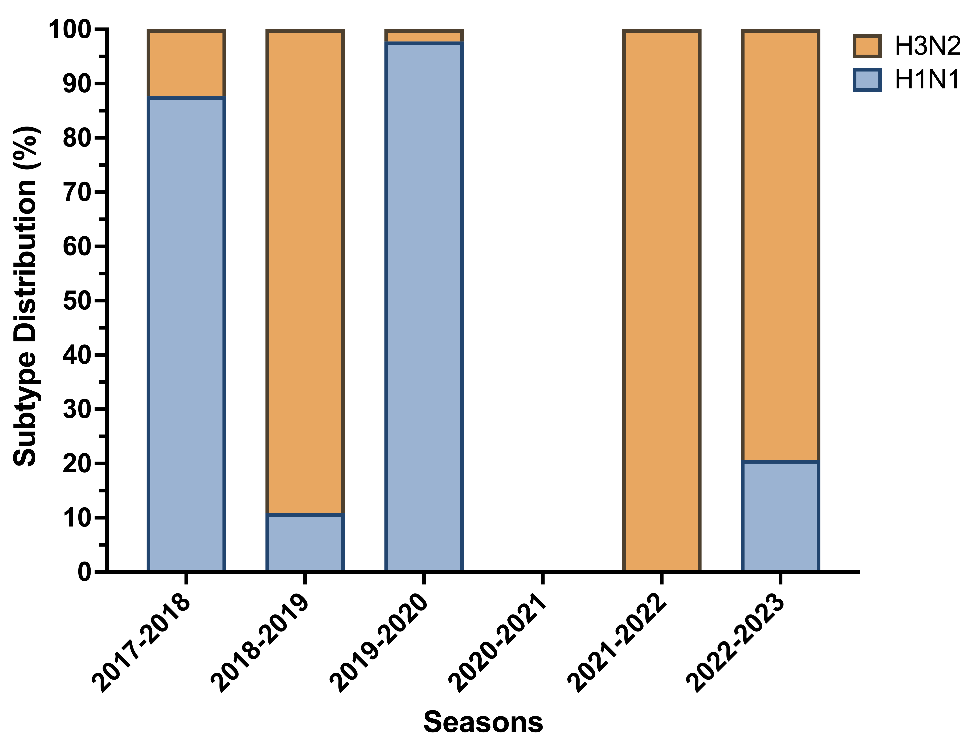
**

**Figure S2.** Subtype distribution of IAVs in Türkiye. The H1 and H3 subtype distribution of IAVs in Türkiye between 2017-2023 was obtained from the publicly available weekly influenza reports that were prepared by the General Directorate of Public Health, Ministry of Health in Turkish (https://grip.saglik.gov.tr/tr/haftalik-influenza-raporu). Since no IAV data were available for the 2019-2020 season, the corresponding column was left empty. Subtypes were color-coded: H3N2 in orange and H1N1 in blue.

**
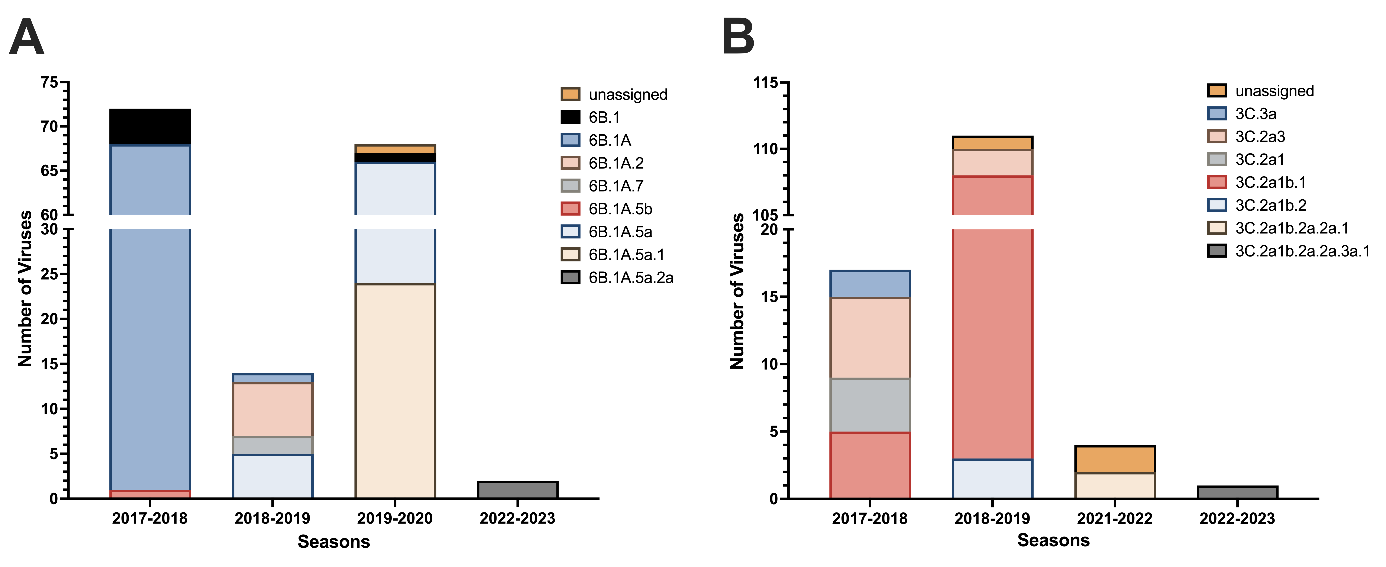
**

**Figure S3.** HA clade distribution of IAVs in Türkiye. The HA clades of H1 (A) and H3 (B) IAVs in Türkiye between 2017-2023 were extracted from publicly available metadata obtained from GISAID. Clades were color-coded: unassigned in orange, 6B.1 in black, 6B.1A in dark blue, 6B.1A.2 in pink, 6B.1A.7 in light gray, 6B.1A.5b in salmon, 6B.1A.5a in light blue, 6B.1A.5a.1 in light brown, and 6B.1A.5a.2a in dark gray for H1N1 viruses, unassigned in orange, 3C.3a in dark blue, 3C.2a3 in pink, 3C.2a1 in light gray, 3C.2a1b.1 in salmon, 3C.2a1b.2 in light blue, 3C.2a1b.2a.2a.1 in light brown, and 3C.2a1b.2a.2a.3a.1 in dark gray for H3N2 viruses.
